# Delirium Tremens

**Published:** 1874-03

**Authors:** D. McMarshall

**Affiliations:** Hopper’s Mills, Ill.


### DELIRIUM TREMENS.

A PAPER READ AT 17TH SEMI-ANNUAL MEETING OF THE MILITARY TRACT MEDICAL SOCIETY, BY D. McMARSHALL, M.D., HOPPER'S MILLS, ILL.

THE account given of the character, phenomena, causes and treatment of delirium tremens, is, upon the whole, so very judicious, that it would, scarcely appear to demand any other comment than one of general approval. But as there exists a very decided difference of opinion among American practitioners in regard to the proper management of the disease, a few words upon this point may not be improper.

Four different plans of treatment have been recommended, and the result of their extensive employment, for a series of years, have been adduced by their respective advocates in evidence of the superior efficacy of each. One practitioner cures all, or nearly all, his cases, by repeated emetics; another, by the free exhibition of alcoholic drinks; and a third by opiates, in free doses, continued at short intervals, until sleep is procured; while a fourth considers neither excitants proper, nor opiates necessary, but simply a state of tranquility in a quiet and darkened chamber, with perhaps an emetic to unload the stomach, in the commencement of the attack, and some gentle cathartic to keep the bowels open, and, when the stomach will retain it, a light and easily digested diet.

The opiate practice is the one in favor of the superior efficacy of which we have the most imposing evidence, and it is unquestionably the one that will, in the majority of cases, when judiciously and cautiously managed, the most promptly and effectually remove the symptoms of the disease. That the opiate practice has been abused, we are perfectly aware. Under the supposition that opium, to any extent that may be requisite to induce speedy sleep, can be administered in delirium tremens with perfect safety, we have cause to fear that a state of coma has, in more than one instance, been induced, from which the patient has never awoke. We have never been in the habit of administering large doses of opium, and have usually combined each dose with an equal quantity of camphor, and about half a grain of ipecacuanha, on young, robust and plethoric subjects. We believe that depletion, in some way, should be resorted to.

That there are many cases of delirium tremens in which a perfect recovery may be effected without the administration of opium, or of any stimulant, is very certain; but our experience has taught us that, when the disease occurs in confirmed inebriates with a broken down constitution, and in whom there is almost complete destruction of the proper functions of the digestive organs, almost the only means by which it can be certainly and promptly arrested is opium, administered in moderate doses, at short intervals.

The treatment of delirium tremens by alcoholic drinks, while we can have no doubt of its very general efficacy, is attended with an evil of too serious a character to permit us to give to it, under any circumstances, our sanction. It cannot fail, we are persuaded, to confirm the patient in his intemperate habits, and thus render him liable to a renewal of the disease after a short interval.

That it is not the only successful treatment, we are convinced from ample experience. In the practice of our preceptors, as well as our own, which has extended now beyond a quarter of a century, or, I may say, a half, we have had sufficient opportunities for testing the value of the opiate practice in this disease, and have seldom been disappointed in its effects. We do not say that the patient will invariably recover under it. There are cases in which, from the condition of the patient's system, the complication of the stimulant delirium with serious disease of the brain or other important organs, death is inevitable, under any plan of treatment. We believe, however, that in the general run of cases, the success of a properly conducted opiate treatment will equal that of any other; while, in the old, broken-down drunkard, it, or the stimulant practice, is the only one upon which any dependence can be placed. Of the emetic treatment as recommended by Dr. Klapp, we cannot, it is true, speak from experience, having never tried it.

I will here report a case treated during the year 1873, by myself. The patient is a resident of Carman Station, where this form of disease has been prevailing to quite an alarming extent. On April 16, 1873, I was summoned to attend Mr. C.; found him suffering from delirium tremens; pulse soft and regular; skin moist; nerves pretty badly convulsed. Gave two and one-half grains opium, and left valerianate of ammonia, in drachm doses. The opiate produced the desired effect, complete repose. On my return, the day following, I found patient quite comfortable; continued the valerianate of ammonia. Saw him on the following day; found patient quite well. Did not see patient again until I was summoned to see him on the 7th day of October last; found him suffering from convulsions, threatening his destruction in a short time, if not arrested; pulse quick and hard; skin dry; convulsions following each other in quick succession. I proceeded to open a vein in the arm, and succeeded in getting a full stream of blood. The patient being strong and plethoric, I allowed the blood to flow till quite two quarts were abstracted; found the pulse soft and regular; patient felt quite relieved, and I supposed that the case was cured, but found, in the course of an hour, that the convulsions returned just as violent as before. I took the compress from the arm, and allowed the blood to flow again to the amount of one pint, when the convulsions disappeared. I then administered to patient sixty grains hydrate chloral, as near as I could guess. Patient slept quietly all night, and on my return the next day found him quite comfortable. Put him on valerianate of ammonia, as before; saw him the day following, and found him entirely recovered, except that he was weak. Did not see patient again till November 7, when I was again summoned to visit Mr. C., and found him bleeding profusely at the nose, from the effect of a fall during the fit he had, just previous to my arrival. I

found no necessity for blood-letting on this occasion, the pulse being soft and regular. I administered hydrate chloral, as before, which produced quietude and sleep, as before; left valerianate of ammonia, in drachm doses, as before, with about the same effect.

I have not seen patient since, professionally, but have seen him attending to his business without any apparent inconvenience.

